# Cassava Breeding II: Phenotypic Correlations through the Different Stages of Selection

**DOI:** 10.3389/fpls.2016.01649

**Published:** 2016-12-15

**Authors:** Orlando Joaqui Barandica, Juan C. Pérez, Jorge I. Lenis, Fernando Calle, Nelson Morante, Lizbeth Pino, Clair H. Hershey, Hernán Ceballos

**Affiliations:** ^1^International Center for Tropical Agriculture, Apartado Aéreo 6713Cali, Colombia; ^2^Corporación Colombiana de Investigación AgropecuariaSanta Marta, Colombia

**Keywords:** early selection, epigenetic effects, experimental error, quality of planting material, repeatability

## Abstract

Breeding cassava relies on a phenotypic recurrent selection that takes advantage of the vegetative propagation of this crop. Successive stages of selection (single row trial–SRT; preliminary yield trial–PYT; advanced yield trial–AYT; and uniform yield trials UYT), gradually reduce the number of genotypes as the plot size, number of replications and locations increase. An important feature of this scheme is that, because of the clonal, reproduction of cassava, the same identical genotypes are evaluated throughout these four successive stages of selection. For this study data, from 14 years (more than 30,000 data points) of evaluation in a sub-humid tropical environment was consolidated for a meta-analysis. Correlation coefficients for fresh root yield (FRY), dry matter content (DMC), harvest index (HIN), and plant type score (PTS) along the different stages of selection were estimated. DMC and PTS measured in different trials showed the highest correlation coefficients, indicating a relatively good repeatability. HIN had an intermediate repeatability, whereas FRY had the lowest value. The association between HIN and FRY was lower than expected, suggesting that HIN in early stages was not reliable as indirect selection for FRY in later stages. There was a consistent decrease in the average performance of clones grown in PYTs compared with the earlier evaluation of the same genotypes at SRTs. A feasible explanation for this trend is the impact of the environment on the physiological and nutritional status of the planting material and/or epigenetic effects. The usefulness of HIN is questioned. Measuring this variable takes considerable efforts at harvest time. DMC and FRY showed a weak positive association in SRT (*r* = 0.21) but a clearly negative one at UYT (*r* = −0.42). The change in the relationship between these variables is the result of selection. In later stages of selection, the plant is forced to maximize productivity on a dry weight basis either by maximizing FRY or DMC, but not both. Alternatively, the plant may achieve high dry root yield by simultaneously attaining “acceptable” (but not maximum) levels of FRY and DMC.

## Introduction

Formal cassava breeding began in the 1930s in eastern Africa and in Brazil but these were isolated programs, generally small, and discontinuous. The creation of the two international centers that work on cassava breeding IITA (International Institute of Tropical Agriculture) and CIAT in the late 1960s, coincided with a rapid expansion of cassava research programs on a national level in Asia, Latin America, and Africa (Westwood, 1990; Gonçalves Fukuda et al., 2002). Several reviews on cassava breeding have been published over the years (Hahn et al., 1979; Byrne, 1984; Jennings and Hershey, 1985; Gonçalves Fukuda et al., 2002; Jennings and Iglesias, 2002; Ceballos et al., 2004, 2012; Kawano and Cock, 2005). The cassava-breeding project at CIAT was created targeting low input conditions in less favorable environments to alleviate the poverty of small farmers through income generation (Kawano and Cock, 2005). From its initiation, CIAT worked in close partnership with national programs and its sister center IITA based in Nigeria.

When cassava plays a food security role, often in marginal agricultural conditions, many traits need to be taken into account: timing of harvest, suitability for intercropping and/or leaf production, taste, bitterness, processing amenability, cooking quality, and even some traits that may just have a role as morphological markers, such as petiole or shoot color, leaf lobule shape or branching characteristics (Benesi et al., 2010). Farmers' participatory approaches have been developed and are ideally suited for addressing these requirements (Gonçalvez Fukuda et al., 2000; Gonçalvez Fukuda and Saad, 2001; Manu-Aduening et al., 2006). However, good opportunities also emerged for more commercial, market-oriented production of cassava particularly in Southeast Asia. CIAT, therefore, took the strategic decision to establish an applied breeding center in Thailand (Kawano and Cock, 2005). In these conditions, centralized, on-station breeding efforts proved to be extremely successful and the results help to explain the current high productivity of cassava in Asia (Hershey et al., 2001; Kawano, 2003; Howeler, 2012). Cassava in Asia developed for the production of starch and dried root chips (the latter were initially exported in large volumes to Europe to be used as source of energy in the composition of animal feed and later for ethanol production in China). Breeding could concentrate on fewer traits (fresh root production, dry matter content and optimum harvest index) and production took place without much pest or disease pressure. It is important for cassava breeders to have a clear understanding of the target farming conditions their cultivars need to be bred for, as well as their main end-uses.

In recent years increasing attention has been paid to reduce the environmental impact of cassava cultivation and processing toward more eco-efficient practices (Hershey and Neate, 2013). Throughout the years there has been a gradual shift of emphasis from the early target on subsistence farming conditions into commercial cultivation and higher-inputs for cassava. In the process breeders became aware that it is difficult, if not impossible, to develop cultivars that could be outstanding simultaneously for fresh consumption (e.g., food security) and the different processing industries (e.g., starch). Therefore, the idea of breeding for multi-purpose varieties gradually gave rise to the need to breed for specific end uses.

Cassava is bred through phenotypic recurrent selection, as it is frequently the case for other clonally propagated crops (Burton, 1992; Grüneberg et al., 2009; Lebot, 2010; Quero-García et al., 2010). Because of the low multiplication rate of cassava, it takes several years to have enough planting material available for replicated multi-location evaluations (Kawano et al., 1998; Jennings and Iglesias, 2002). A typical selection cycle requires 2 years to produce the botanical seeds of planned crosses and six consecutive years of field evaluation. Early phenotypic evaluations are based in non-replicated trials grown in a single location. Critical decisions are taken through this lengthy process and cassava breeders try to conciliate the need to reduce the large number of genotypes in the early stages of selection with the awareness that selection, based in trials without replications is prone to large experimental errors.

Before the year 2000, selection at early stages was done visually and the only information available was if a genotype was or not selected. No phenotypic information was available. This was an efficient way to process large segregating populations. Important advances were made improving plant type architecture and defining the breeding populations for different target environments. A decision was made in 2000 to change the strategy and start registering data of all genotypes evaluated, regardless they were selected or not. For about 15 years, therefore, CIAT has conducted the same phenotypic recurrent selection process. A large amount of information has been generated and can be used to relate the information generated by the germplasm reaching the multi-location stage of evaluation with all the previous stages of selection. An article has already been published using the same data (Ceballos et al., 2016). This knowledge is crucial for determining which variables are more efficiently selected for at different stages of selection. The objectives of this study were: (i) to consolidate phenotypic data from 14 years of trials; (ii) analyze the relationship for each variable along the lengthy selection process; (iii) quantify associations between different variables at each stage of selection and (iv) use the information generated to suggest changes in the phenotypic recurrent selection approaches currently used in cassava breeding at CIAT.

## Materials and methods

Data from evaluation trials conducted since 2000 through 2013 was recovered. This study concentrates only on data from the sub-humid environment, the most important cassava growing region in Colombia and worldwide.

### Breeding objectives and selection criteria

A wide range of breeding objectives is needed given the diversity of uses and environments for cassava production. However, few are widely accepted by every breeding program as key traits that must be taken into consideration: high fresh root yield (FRY); high and stable dry matter content (DMC); adequate plant architecture and resistance to pest and diseases which at CIAT is integrated in a plant type score (PTS, ranging from 1 = clearly better than the average to 5 = clearly worse than the average). Selection for high FRY has limitations because of its low heritability (particularly in early stages of selection with small, non-replicated plots). As an alternative, indirect selection for harvest index (HIN) was used for many years. This strategy took advantage of the higher heritability for HIN and the acceptable correlations with FRY (Kawano et al., 1998; Kawano, 2003).

A selection index (SIN) is generally used integrating these four relevant variables, assigning them an arbitrary weight (W_n_) established by the breeder (Ceballos et al., 2012):

$$\begin{array}{l} \text{SIN}=\left({\text{FRY}*\text{W}}_{1}\right)+\left({\text{DMC}*\text{W}}_{2}\right)-\left({\text{PTS}*\text{W}}_{3}\right)+\left({\text{HIN}*\text{W}}_{4}\right) \end{array}$$

In the case of PTS desirable target is a lower score. Therefore, a negative sign is assigned to the respective term in the SIN equation. Typical weights used over the years have been W_1_ and W_2_ = 10; W_3_ = 5; and W_4_ = 3.

### Evaluation scheme and selection process

Botanical seeds, obtained from crossing outstanding (heterozygous) progenitors, are germinated and seedlings grown in a greenhouse until they are transplanted to the field 2 months after germination. Over the years, botanical seeds from at least 297 progenitors were germinated and evaluated in the field. The seedling plants (F_1_s) were grown for 10–11 months and then plants were selected and harvested. The only selection criterion was the capacity of the plant to produce eight vegetative cuttings for the following stage of selection. Doing this selection, by default, eliminated plants susceptible to thrips. F_1_ populations were grown in Palmira (location of CIAT headquarters) which offers ideal growing conditions and availability of irrigation. Planting material from the selected seedling plants were then shipped to the sub-humid environment, which is the most important cassava growing environment in Colombia and elsewhere. The phenotypic recurrent selection continued in the target environments as described below.

#### Clonal evaluation trials or single row trials (SRTs)

This is the first selection for agronomic performance and takes place in each target environment. These are large trials (≈1–2 ha) grown in a single location. Each genotype is represented by eight plants grown in a single row. Stratified selection is used to reduce the error due to environmental variation (Gardner, 1961; Ceballos et al., 2004, 2012). Selection is therefore exerted within each stratum. About 1000–2500 genotypes are evaluated at this stage and about 15% are selected for the next stage of selection. More than 20,000 genotypes were evaluated in SRT and reported in this study (9108 from full-sib families and 11,221 from half-sib families). A total of 1038 full- or half-sib families were involved. An important feature of this type of trial is that planting material originated in the nearly ideal environment of Palmira.

#### Preliminary yield trials (PYT)

They are also planted in a single location. Each genotype is represented in three repetitions. Plots in each repetition have two rows with five plants per row. Three or four PYTs (with 50–100 genotypes each) are planted, thus reducing the size of trials. Planting of these trials is done in a special way to avoid competition among genotypes with contrasting plant architecture. Row spacing is reduced to 0.8 m (from the standard 1 m). Each genotype is planted in two neighboring rows with five plants each (for the 10-plant plot mentioned above). An empty row is left between plots with different genotypes. Plant-to-plant distance within the row is reduced to 0.8 m. This approach allows growing the evaluations with a plant density close to the standard 10,000 plants ha^−1^, while reducing competition among different genotypes and favoring competition among plants from the same genotype. Over the 14 years of evaluation described in this study, 2866 genotypes were evaluated in PYTs. An important feature of this type of trial is that planting material originates, for the first time, in the target sub-humid environment. This is also the case for the subsequent trials.

#### Advanced yield trials (AYT)

Plots at this stage of selection are larger than those from previous stages with four (or five) rows and five plants per row. Three replications are used in a single location. The six (or nine) central plants are harvested to generate the data used in the selection process. The surrounding 14 (or 16) plants in the periphery of the plot are used as source of planting material when required as they can be left standing in the field. Since only the central plants of the plot are harvested there is no need for a special planting arrangement to reduce competition between different genotypes. Plant density for these trials, therefore, is the normal one (10,000 plants ha^−1^), with 1 m between rows and 1 m spacing between plants within a row. The selected clones from the different experiments at the PYT stage are merged to produce a single AYT with 50 to 80 genotypes. A total of 615 genotypes were evaluated in AYT and reported in this study.

#### Uniform yield trials (UYT)

This is the final stage in the evaluation and selection process. Plot size, number of repetitions and planting arrangement is the same as those for AYTs. UYTs are planted for two consecutive years in 3–6 locations. Typically, UYTs will have 10–20 experimental clones and 5–8 local or commercial checks. Depending on the performance of the experimental clones compared with those of the checks there may be an official release when experimental clones show an outstanding performance. An interesting step taken at this stage is that planting material of the most promising clones is shared with key farmers for semi-commercial evaluation. In general, cultivars are released only after successful performance (according to the farmers' criteria) in these semi-commercial evaluations (0.5–1 ha). Farmers and end-users are often invited to participate during the harvest of AYTs and UYTs and their opinion is informally incorporated into the selection process. Planting material of most of the genotypes reaching the UYT are brought back to Palmira and incorporated to the crossing nurseries to start a new recurrent cycle. Only 114 genotypes were evaluated in UYT during the time period reported in this study.

### Data analysis

Data from trials grown in different years were consolidated into a large Excel file (Microsoft, 2015)^1^ with more than 30,000 data points. Data was not always available for all variables considered. Depending on the trait, data from 19,498 to 20,379 genotypes evaluated in SRT is available (Table 1). As stated above, the number of genotypes was gradually reduced with the successive replicated trials. Averages for each of the 2866, 615, and 114 genotypes evaluated, respectively, in PYT, AYT, and UYT were estimated. In some cases, however, few data points were missing or were eliminated during the data curation process, which explains slight variation in degrees of freedom for different comparisons.

**Table 1:** **Averages for different variables estimated at the successive stages of phenotypic evaluation of cassava adapted to the sub-humid environment**.

**Parameter**	**Plant type score (1–5)**	**Harvest index (0–1)**	**Dry matter content (%)**	**Fresh root yield (t ha^−1^)**
**SINGLE ROW TRIAL (SRT) BEFORE SELECTION**				
Average	3.07	0.47	30.29	17.85
Standard Deviation	0.99	0.12	4.97	9.17
CV (%)	32.24	25.44	16.40	51.36
*n*	20379	19552	20324	19498
**SINGLE ROW TRIAL (SRT) AFTER SELECTION**				
Average	2.46	0.55	32.10	27.69
Standard Deviation	0.89	0.08	4.49	9.85
CV (%)	35.93	15.30	13.99	35.57
*n*	2817	2740	2865	2690
**PRELIMINARY YIELD TRIAL (PYT)**				
Average	2.89	0.51	30.78	19.44
Standard Deviation	0.79	0.11	3.85	8.43
CV (%)	27.39	21.18	12.49	43.37
*n*	2866	2866	2866	2866
**ADVANCED YIELD TRIAL (AYT)**				
Average	2.73	0.57	33.67	23.80
Standard Deviation	0.57	0.07	2.51	6.27
CV (%)	21.03	11.92	7.46	26.35
*n*	603	615	615	615
**UNIFORM YIELD TRIAL (UYT)**				
Average	2.70	0.58	33.57	24.00
Standard Deviation	0.47	0.06	1.77	3.44
CV (%)	17.30	10.21	5.28	14.33
*n*	114	114	114	114

#### Evolution of performance across the different stages of the selection process

The available data offers a unique opportunity to assess how the average performance of genotypes evolves from the unselected SRT through the successive PYT, AYT, and UYT steps. Average performance along with other statistical parameters were estimated for each stage and across the different years.

#### Repeatability for each variable along the different stages of the selection process

One of the questions that this study aims answering is the reliability of early measurement for different traits (PTS, HIN, DMC, and FRY). For each variable, correlation coefficients were estimated to assess the relationships for data taken at different types of trial for each genotype. Linear correlations were estimated using the following formula:

(1) $$\begin{array}{l} r=\frac{{\overset{\text{n}}{\sum }}_{\text{i}=\text{1}}\left({\text{x}}_{\text{i}}-\text{X}\right)\;\left({\text{y}}_{\text{i}}-\text{Y}\right)}{\surd {\overset{\text{n}}{\sum }}_{\text{i}=\text{1}}{\left({\text{x}}_{\text{i}}-\text{X}\right)}^{\text{2}}\surd {\overset{\text{n}}{\sum }}_{\text{i}=\text{1}}{\left({\text{y}}_{\text{i}}-\text{Y}\right)}^{\text{2}}}=\frac{\sum {\text{x}}_{\text{i}}{\text{y}}_{\text{i}}}{\surd \left(\sum {{\text{x}}_{\text{i}}}^{\text{2}}\right)\left(\sum {{\text{y}}_{\text{i}}}^{\text{2}}\right)} \end{array}$$

where x_i_ is the observation for a given trait and genotype in one kind of trial (and X is the average across genotypes for that trial), and y_*i*_ is the observation for the same trait and genotype but in a different type of trial (and Y is the respective average). Correlations and their significance were estimated using SAS (2008). In addition, Spearman's rank correlation analysis was performed. This is the correlation coefficient between the ranked values (Snedecor and Cochram, 1980).

The correlations between SRT, PYT, or AYT and the multi-location UYTs were based on phenotypic data from different number of genotypes. In these comparisons, when trials involved more than one replication/location, correlations were made on the average of the genotype across replications. Similarly, in the case of UYT the data used were the averages across locations and years.

#### The value of HIN as indirect selection for FRY

Another question that this study aims answering is the potential of HIN (measured early in the selection process) as indirect selection for FRY (in later stages of selection). Pearson's correlation analyses between HIN (in SRT, PYT, and AYT) and FRY (at UYT) were estimated using SAS (2008).

#### Possibility of simultaneous progress for more than one trait through SIN

Successful varieties require the combination of more than one trait to satisfy farmers' expectations. In many cases two desirable traits may be negatively associated. The association among different traits with FRY within each type of trial was therefore estimated also using linear correlation coefficients. It was also particularly interesting to determine if DMC shows any type of association with FRY. Correlations and their significance were estimated using SAS (2008).

## Results

The same data presented here was used for a “sister” article attempting to identify factors in SRT that could predict probabilities of genotypes reaching the UYT stage (Ceballos et al., 2016). All available data has been used here for each analysis to maximize the degrees of freedom and avoid any undesirable bias in the analysis. Degrees of freedom in this study may be, therefore, slightly different from that previous study based on the same dataset.

### Evolution of performance across the different stages of the selection process

Table 1 provides a summary with the averages for each variable across the different types of trial. Data from at least 20,379 genotypes evaluated at SRT during the past 14 years is available. A unique feature of this dataset is that information of each genotype is available regardless they were selected or not. For many plots data could not be obtained because of missing plants or, in the case of FRY negligible yield (population size for unselected SRT = 19,498) or mistakes during data uploading. The average of selected materials at SRT is also provided in Table 1 and, as expected, resulted in better averages, particularly for FRY (27.69 vs. 17.85 t ha^−1^). The sample size in PYT was always 2866, whereas the sample size for the selected materials at SRT is slightly smaller. In many cases genotypes were selected even though data for a particular trait was not available. A remarkable trend that can be observed in Table 1 is the poorer performance at PYTs, compared with the averages of the selected materials at SRT. It shows lower FRY (19.44 vs. 27.69 t ha^−1^) and DMC (30.78 vs. 32.10%). PTS was also worse (2.89 vs. 2.46) in PYT compared with data from the same genotypes at SRT. A lower PTS score is desirable. HIN showed a similar trend (0.51 vs. 0.55). For every variable averages were worse in PYT than in any other subsequent trial.

### Repeatability for each variable along the different stages of the selection process

Table 2 presents the linear correlation coefficients for each variable. These correlations relate the phenotypic data for the same trait and the same genotype evaluated in different stages of the selection process. Spearman's rank correlation analysis was also performed. However, results are not presented as the two types of correlations provided basically the same information. For each variable presented in Table 2, different data sets were used to estimate the correlations. In the top three rows for each variable, correlations between SRT with PYT, AYT, or UYT are presented. Below these rows the correlations between PYT with AYT or UYT are provided. Finally, the last row presents correlations between AYT and UYT. Sample sizes for each type of comparison are also provided.

**Table 2:** **Linear correlations coefficients for each variable evaluated in different stages of the selection process (within parenthesis number of observations used to estimate the correlation)**.

**Type of comparison**	**Plant type score 1–5**	**Harvest index 0.0–1.0**	**Dry matter content %**	**Fresh root yield t ha^−1^**
SRT vs. PYT	0.32^**^	0.42^**^	041^**^	0.12^**^
	(2817)	(2740)	(2865)	(2690)
SRT vs. AYT	0.29^**^	0.36^**^	0.37^**^	0.32^**^
	(590)	(591)	(614)	(579)
SRT vs. UYT	0.35^**^	0.17^NS^	0.37^**^	0.29^**^
	(114)	(114)	(114)	(114)
PYT vs. AYT	0.45^**^	0.37^**^	0.43^**^	−0.02^NS^
	(603)	(615)	(615)	(615)
PYT vs. UYT	0.34^**^	0.32^**^	0.70^**^	0.29^**^
	(114)	(114)	(114)	(114)
AYT vs. UYT	0.72^**^	0.46^**^	0.53^**^	0.63^**^
	(114)	(114)	(114)	(114)

** Significant at the 1%;

NS *Non-significant*.

The highest correlations were observed for the comparison between AYT and UYT for every variable, except DMC (Table 2). Correlations involving comparisons with SRT tended to be the lowest. Because of the large sample sizes most coefficients are statistically significant, even though some of them have indeed small magnitude. For example, the coefficient for FRY measured in SRT and PYT (*n* = 2690) was only 0.12, yet highly significant (*P* < 0.01). The only non-significant correlations were for HIN (between SRT and UYT) and FRY (between PYT and AYT). An interesting pattern can be observed in correlations involving SRT (first three rows in Table 2). For FRY, PTS, and DMC there is not a clear tendency for the coefficients as SRT are contrasted successively with PYT, AYT, and UYT. In the case of HIN, on the other hand, the correlation coefficients decreased consistently 0.42; 0.36 and 0.17 for SRT vs. PYT; AYT and UYT, respectively.

### The value of HIN as indirect selection for FRY

Table 3 presents the correlation coefficients between HIN and FRY in different type of trials. It is particularly relevant and disappointing the low and non-significant value (*r* = 0.14) between HIN in SRT and FRY in UYT. In fact, this value was lower than that of FRY at SRT vs. UYT (*r* = 0.29) presented in Table 2. The justification of using HIN in SRT was based on the evidence that it was a better predictor for FRY in UYT than FRY itself (Kawano et al., 1998; Kawano, 2003). Data presented in this study would somewhat contradict these earlier findings.

**Table 3:** **Usefulness of HIN as indirect selection in early stages of evaluation for increased FRY in later stages of selection**.

**Type of comparison (sample size)**	**Harvest index (0.0–1.0)**
HIN in SRT vs. FRY in PYT (2740)	0.12^**^
HIN in SRT vs. FRY in AYT (591)	0.11^*^
HIN in SRT vs. FRY in UYT (114)	0.14^NS^
HIN in PYT vs. FRY in AYT (615)	0.27^**^
HIN in PYT vs. FRY in UYT (114)	0.20^*^
HIN in AYT vs. FRY in UYT (114)	−0.11^NS^

*Pearson's correlation coefficients of HIN with FRY assessed in the successive evaluation stages for cassava breeding at CIAT*.

*,** Significant at the 5 and 1% level, respectively.

NS *Non-significant*.

### Possibility of simultaneous progress for more than one trait through SIN

The justification for using a selection index takes into consideration that farmers require varieties that simultaneously satisfy few key traits. Only then varieties would be adopted.

The traits to be taken into account will vary depending on the end-use(s) of the varieties as well. It is well-recognized, however, that in some cases desirable traits may be negatively correlated. Data presented in Table 4 aims at understanding the relationship between different variables with FRY within the same trials (phenotypic correlations).

**Table 4:** **Pearson's correlation coefficients of different variables with FRY assessed in the same trial (within parenthesis number of observations used to estimate the correlation)**.

**Trial type**	**DMC**	**PTS**	**HIN**
SRT^a^	0.21^**^ (19455)	−0.30^**^ (19498)	0.47^**^ (19498)
PYT^b^	−0.13^**^ (2866)	−0.23^**^ (2866)	0.02^NS^ (2866)
AYT^b^	−0.14^**^ (615)	−0.09^*^ (603)	0.12^**^ (615)
UYT^b^	−0.42^**^ (114)	−0.04^NS^ (114)	0.24^*^ (114)

a *Correlations on a plot basis*.

b *Correlations based on averages of genotypes across reps (and locations/years in UYT)*.

*,** Significant at the 5 and 1% level, respectively.

NS *Non-significant*.

There was an interesting trend regarding the association between DMC and FRY (Table 4). In SRT, the coefficient was positive (*r* = 0.21) but gradually and consistently this relationship became increasingly negative in the successive stages of selection. In UYT the correlation was clearly negative (*r* = −0.42). Another trend that can be observed in Table 4 is the negative association between PTS and FRY. This is to be expected as the scores for PTS range from 1 (excellent phenotype) through 5 (very undesirable plant type). The magnitude of the negative correlation coefficients consistently shrinks in successive stages of selection (from −0.30 in SRT down to a non-significant −0.04 in UYT). This must also be the result of selection. Correlations between HIN and FRY in different types of trial can also be found in Table 4. In a way it is expected that these variables should have an association since FRY is the numerator in the equation to estimate HIN. There is a clear contrast, however, for the association between these variables in SRT (*r* = 0.47) compared with that at PYT (*r* = 0.02). The correlation coefficients then improve in AYT (*r* = 0.12) and UYT (*R* = 0.24). There is an unexpected result for the correlations in PYT which may be related to the poor performance at PYTs described in Table 1.

## Discussion

The phenotypic recurrent selection in cassava has the advantage that the same cloned genotypes can be evaluated and selected many times in different locations and growing seasons. The gradual selection, through four different stages (SRT, PRY, AYT, and UYT) allows the selection of genotypes that have shown outstanding performance consistently. However, the process does not guarantee that really the best germplasm is selected at each stage. This study quantified the reliability of selections for the different traits in the successive evaluation stages. There are several confounding effects that are expected to weaken the correlation coefficients.

Because of the length of each recurrent selection cycle and the limitations of planting materials in early stages, it is important to maximize the possibilities of selecting the best genotypes early in the process. An analysis of the relationship of different variables measured successively in the four stages of the selection process is therefore, relevant. The present study covers 14 years of data and therefore conclusions are considerably more robust than previous reports (Kawano et al., 1987, 1998; Morante et al., 2005). Early stages of selection are based on a single location in Santo Tomás (Atlántico Department, Colombia), whereas uniform yield trials are planted in several locations scattered in several Departments in Colombia (Atlántico, Bolivar, Sucre, Magdalena, Córdoba, and Cesar) and usually for more than one season.

The environmental variation from year to year is an important component of the genotype-by-environment interaction. Year to year variation is particularly detrimental for DMC because the arrival of the rains is not constant and is becoming increasingly unpredictable (as a result of climate change). In some cases rains arrived earlier than usual and in these cases harvest took place after the plants in the field had begun sprouting. When this happens, DMC is drastically reduced (Ceballos et al., 2011). However, this reduction is not uniform across genotypes therefore resulting in genotype-by-environment interaction. Year to year variation will also affect PTS as temperature and rainfall will affect not only plant architecture but also the impact of pest and diseases. In addition there is a geographic variation as trails may be grown in one farm 1 year and in a different farm the following year. UYT, in particular, are grown in a much wider geographic region.

**Figure 1:**
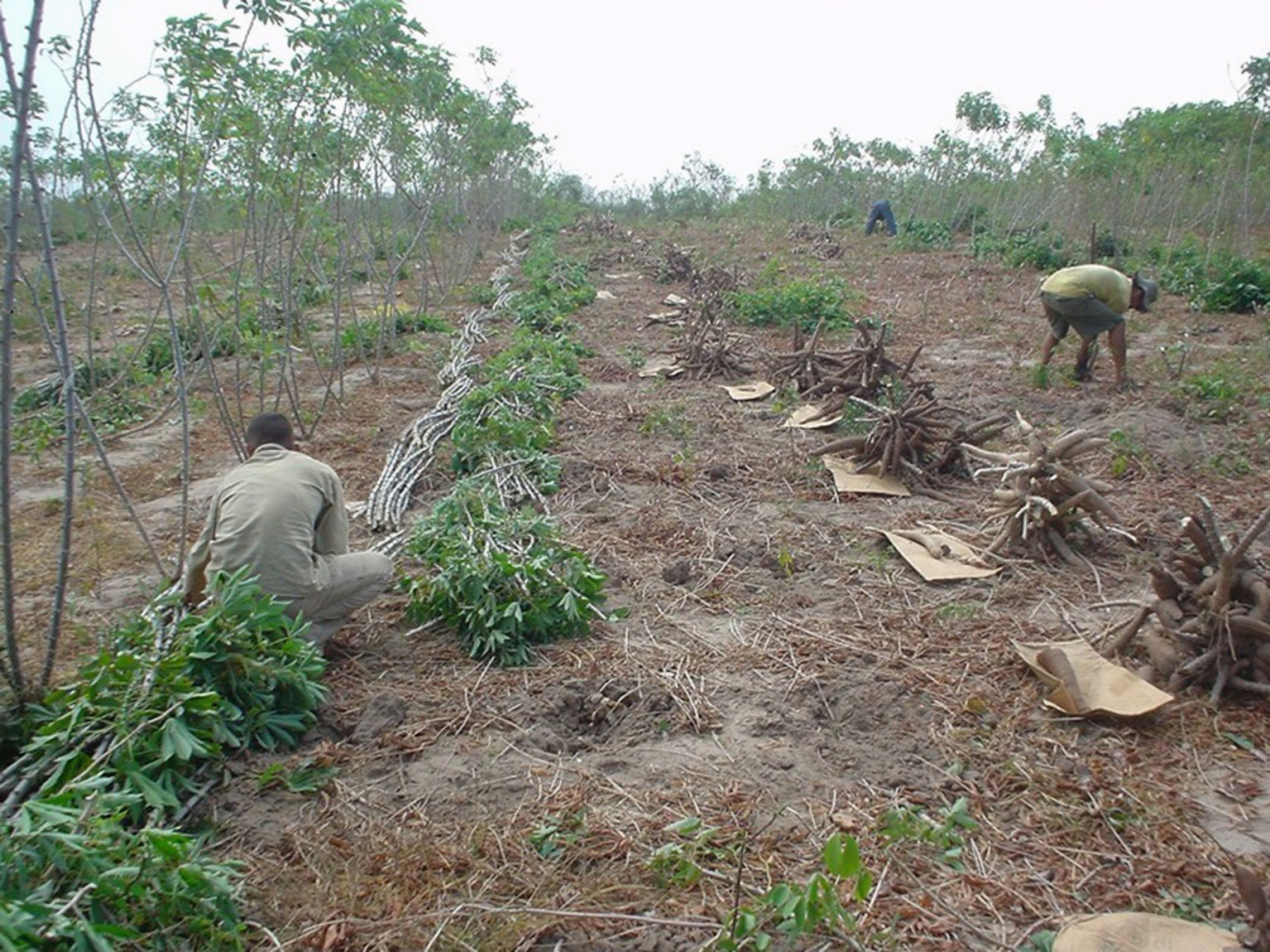
**Illustration of a typical single row trial**. The plant in the front of the row is kept standing until the collection of the planting material of selected genotypes. These trials, in particular, are large (covering 1–2 hectares) which, together with the lack of uniformity of farmers' fields, make the selection at this stage prone to large experimental errors.

In addition to all the effects mentioned above, the experimental errors for each trial must also be considered. These errors tend to be very large in SRT because of the size of the experiments (1–2 ha) and the natural variation of on-farm evaluations for many of the typical target environments for cassava (Figure 1). Missing plants also contribute to the experimental error. However, a correction for missing plants is now being implemented (Pérez et al., 2010), but could not be applied in a large proportion of the data presented herein.

### Evolution of performance across the different stages of the selection process. the “stabilization of the genotype” and the relevance of quality of planting material

The poorer than expected performance in PYTs was surprising (Table 1). This is, indeed, a remarkable and robust trend that needs a feasible explanation. Quality of planting material is always an important issue in breeding root and tuber crops. SRTs are planted with stem cuttings coming from Palmira which is a relatively neutral environment for biotic stresses and there is no abiotic constraint affecting their physiological and nutritional status. Seedling plants are not used as source of information and can be harvested just when needed (e.g., after the planting season had clearly began after the arrival of the rains in the target environment). The storage period of planting material used for SRT is thus typically short. However, during the transition from SRT to PYT the germplasm under evaluation starts to suffer from the environmental constraints (water stress) and biotic problems (mites, stem borers, frog skin disease, and to a lesser extent, bacterial blight and super-elongation disease). At harvest time, therefore, the physiological, and nutritional status of the stems harvested in SRT is not uniform but differences will only become apparent the following season (in PYT). On top of this, planting material may need to be stored for few to several weeks until the arrival of the rains. So, a feasible explanation for the poor performance at PYTs is that this is the first trial whose planting material has been produced in the sub-humid conditions. In latter trials averages improve consistently as selected material are those that survive the ordeal related to planting material produced *in situ*. Unusual results for PYT were also noticed for FRY correlations shown in Table 2 and the correlation between HIN vs. FRY in Table 4.

It has always been postulated (but not formally demonstrated) that, as a new genotype is cloned and grown year after year, its performance “*evolves*” reaching an equilibrium with endophytic organisms present in the target environments. Also, as the germplasm faces new conditions in the sub-humid environment, genetic differences in the adaptation of the germplasm to these conditions will translate into differences in the nutritional, sanitary and physiological quality of the planting material. In some cases this “*evolution*” is very negative and quickly the genotypes lose their capacity to produce well. The impact of this effect would be most apparent in the transition from SRT to PYT. It is common (and frustrating) to see materials with very poor performance at PYTs. Breeders wonder how could these genotypes have been selected at SRT and this is, obviously, an important question to answer. A feasible explanation would be the gradual equilibrium reached by different cassava genotypes with the surrounding biotic environment. The so called “*extended genotype*” was described as an interactive cross-organismal genome with potential, exploitable implications by Hale et al. (2014). Another feasible explanation for this situation would be epigenetic effects. It has been already demonstrated that epigenetic effects react specifically to water stress in perennial species affecting, among different traits, bud dormancy (Bräutigam et al., 2013).

The quality of planting material will become even more relevant for cassava in the coming years. One of the most immediate impacts of climate change is the increased uncertainty regarding the time the rainy season begins (Ceballos et al., 2011). Because of this, the storage period of the stems may have to be extended longer than normal with detrimental effect on their sprouting capacity. Higher emphasis, therefore, will be placed to guarantee a uniform and vigorous sprouting of each cutting. SIN will include a term taking this into account.

### Repeatability for each variable along the different stages of the selection process

Correlations for HIN were generally disappointing (Table 2). Correlations involving HIN comparing data from SRT and PYT was relatively high (*r* = 0.42). However, it became gradually weaker in SRT vs. AYT (*r* = 0.36) and SRT vs. UYT (*r* = 0.17). Correlations for HIN at PYT vs. AYT or UYT were more or less stable (*r* = 0.37 and 0.32, respectively). The correlation for HIN between AYT and UYT was only 0.46 (the lowest among the four variables analyzed). These results contrast sharply with those reported by Kawano et al. (1998).

Correlations for DMC were generally high (Table 2) in agreement with Kawano et al. (1987) who reported high heritability values for this trait. Those involving SRT data ranged from 0.37 (SRT/UYT and SRT/AYT) to 0.41 (SRT/PYT). Correlations involving PYT data increased considerably: 0.43 and 0.70, respectively, for PYT/AYT and PYT/UYT. The AYT/UYT correlation (0.53), however, was lower than expected. DMC is usually at its highest value at the end of the dry season. With the arrival of the rains, starch and other nutritional compounds stored in the roots are metabolized to sustain the re-initiation of growth (Kawano et al., 1987). An earlier than normal rainfall will result in a drastic reduction in DMC within a couple of weeks. Typically, average DMC in large segregating trials may be around 32–34%, but if the same trial is harvested 2 weeks after the arrival of the rains the average may go down to 26–28%. Some genotypes, however, show a larger reduction in DMC than others (CIAT, 2001), therefore generating undesirable variation, which ultimately reduces the repeatability of performance. There is a sequence in the harvest of trials which is followed every year. The first harvested trial is the SRT, then PYT and finally AYT and UYTs. The late harvests of AYT and UYTs imply that they have a higher probability to take place after the arrival of the rains with the known result of an increased variability of the results for DMC. Changes in the arrival of the rains (Ceballos et al., 2011) due to climate change may explain the unexpectedly low correlation coefficient for DMC between AYT and UYT.

The correlations for FRY presented in Table 2 illustrate the problems that cassava breeders face. SRT/PYT correlation was negligible (0.12), although it increased for SRT/AYT (*r* = 0.32) and SRT/UYT (*r* = 0.29). Correlations of PYT data with AYT was negligible (−0.02) but improved for PYT/UYT (*r* = 0.29). As expected, however, correlation for FRY between AYT and UYT was relatively high (*r* = 0.63). This is the reason why cassava breeders have always stated that at early stages, selection should focus on traits with higher heritability (e.g., HIN) rather than on FRY (Kawano et al., 1998; Kawano, 2003). The lowest correlations were observed between SRT vs. PYT (0.12) and PYT vs. AYT (−0.02). In both cases data from PYT were involved. Once again there is an unusual result from PYTs.

In general, correlations involving early stages of selection were lower than expected. Feasible explanations need to be explored. The ever confounding effects of genotype-by-environment interaction and errors related to lack of uniformity in large trials, such as SRT (1–2 ha) must be taken into consideration (Figure 1). To overcome some of these problems CIAT stratifies SRTs into smaller plots (Gardner, 1961; Ceballos et al., 2004, 2007, 2010, 2012) therefore reducing the environmental component in the overall phenotypic variance. However, there is one factor in the evaluation scheme that may play a large role explaining the lower than expected correlation coefficients: quality of planting material. This factor has already been described above.

A critical selection takes place at SRTs because the large number of genotypes involved in this type of trial is drastically reduced (only 15% of the clones are selected and ultimately about 12% is planted in PYTs). The correlations at SRT with other types of trial (particularly UYT) is, therefore, particularly relevant. It is in the SRT where mistakes in the selection process are most likely to occur. SRT vs. UYT correlations for DMC and PTS were the highest (*r* = 0.37 and 0.35, respectively). It was surprising, however, to find a higher SRT vs. UYT correlation coefficient for FRY (0.29) than for HIN (0.17). It has been long accepted that selecting in SRT for HIN (rather than for FRY) was more effective for yield improvement (Kawano et al., 1998; Kawano, 2003).

### The value of HIN as indirect selection for FRY

Correlations involving HIN were disappointing particularly when comparisons with UYT data are considered. Repeatability of HIN compared with FRY (Table 2) was marginally better. Moreover, using HIN as an indirect selection criterion (in SRT) for FRY (in UYT) was no better than using FRY itself (Tables 2, 3). The contrast between data presented in this study and earlier data (Kawano et al., 1998; Kawano, 2003) regarding the usefulness of HIN is perplexing. One feasible explanation for these contrasting results was already mentioned above: the need for a “*stabilization*” of the germplasm. In our breeding scheme the origin of the planting material for SRT is different than that for the subsequent trials (PYT, AYT, and UYT) and this may explain some inconsistencies, particularly involving SRT and PYT. Disease and pest pressures in Colombia are higher than those in Thailand (at least for the period reported in Kawano et al. articles). It is tempting to suggest that perhaps cassava has evolved in the past four decades and HIN has been improved to the point that the trait has been “*fixed*” enough and no longer has the impact that it used to have. However, the average HIN in the unselected SRT was 0.47 ± 0.12 (ranging from basically 0.0–0.89) suggesting that there is still ample genetic variation in the breeding population adapted to the sub-humid environment.

Kawano (2003) presented data suggesting that HIN did not change drastically through 20 years of breeding. It was biomass, in fact, what was improved over the years, at least based on released varieties. However, the relative importance of HIN within a given population, according to Kawano, was invariably more important. In our study we pool together 1038 different families. Perhaps for certain families HIN would still be a good predictor for FRY (probably families which show large variation for HIN), but not for other. In Kawano's article the author suggests that “*when a breeding accomplishes a certain level and faces a new challenge, a new operational principle may be necessary*,” Breeding populations at CIAT may have reached that stage where new principles are necessary.

Harvest index (HIN) will remain an important criterion for the selection at the seedling plant stage, along with the capacity to produce 6–8 stem cuttings. However, taking into consideration that measuring HIN requires about half of the efforts in labor during the harvest of SRTs, these results prompted CIAT to take the decision not to measure HIN in future trials. The aboveground biomass will be assessed through PTS, which is considerably less expensive to obtain. Special care will be taken in the future not to reward plant types with excessive aboveground vigor.

### Possibility of simultaneous progress for more than one trait through SIN

Information presented in Table 4 suggests a very dynamic response of segregating material in response to the selection based on SIN. This, in turn is evidence of the efficiency of selection and the “*plasticity*” of cassava populations. The relationship among different variables gradually shifts from one type of trial to another. For example, as selection takes place a weak positive phenotypic correlation between DMC and FRY (0.21) becomes strongly negative (*r* = −0.42). Kawano reported in 1987 positive correlations between DMC and FRY (average of 0.26) similar to the ones observed for SRT in the present study. This trend is not evidence of a physiological association between these variables nor of pleiotropic effects of one variable on the other. It is rather evidence that cassava genotypes attaining maximum dry matter productivity, do so by maximizing DMC or FRY, or reaching acceptable levels simultaneously for both variables. Changes in the relationships between different variables highlight that correlations are not proof of a causation effect, but can help understand how selection modulates the values of the correlations analyzed. The nature of the association between DMC and FRY cannot be properly stated without a clarification in which stage of the selection process this association is being considered. Similar conclusions can be drawn from Kawano et al. (1998). In unselected populations, it can be stated, that DMC and FRY are rather independent. However, in later stages of the selection process both variables show a clearly negative relationship for the reasons explained in this study.

Jennings and Hershey (1985) already stated many years ago that it may prove difficult to maintain high DMC while continuing to increase FRY. That statement is validated by data presented in Table 4. The increasingly negative relationship between these two variables in successive stages of evaluation is probably the result of the selection process. Materials are selected based on a selection index which ultimately rewards those with high dry matter yield (the combination of FRY and DMC). It is within a limited range of high dry matter yield (e.g., clones reaching the UYTs) where genotypes have to choose between strategies based on high FRY, high DMC, or else simultaneously “*acceptable*” (but not maximum) levels for both variables. In other words, it is difficult for a genotype to show simultaneously maximum FRY and DMC levels as this would imply an energy demand (e.g., starch) that the plant cannot satisfy.

Plant type score (PTS) includes plant architecture and reaction to biotic and abiotic stresses. Erect plant architecture is favored as facilitates the harvest, transport and storage of stems that will become the planting material for the following season. Erect types also facilitate cultural practices, such as fertilization (which typically is done twice: 30 and 90 days after planting) and weed control (usually by hand). Late flowering leads to a high height of the first branching and, therefore, it is closely linked with erect plant architecture (Ceballos et al., 2012). PTS is regarded as a moderately high heritability trait. In addition to architecture, plant type score also includes an assessment of the reaction to biotic (particularly mites) and abiotic (water stress) constraints, which are prevalent in the sub-humid conditions. The proportion of stem with leaves still attached is an easy and reliable approach to assess the tolerance to these stresses (Lenis et al., 2006).

In SRT many genotypes will be unfit for the sub-humid environment and both their plant architecture and reaction to the main biotic and abiotic stresses will show large variation. Undesirable plant types are gradually eliminated and in later stages of selection the association between PTS and FRY becomes negligible (Table 4) as all selected materials show acceptable PTS (e.g., the genetic variability for PTS has shrunk). This trend can be the result of a drastic reduction of genotypes with undesirable PTS which is very effective given the good heritability for this trait.

### Concluding remarks

This article describes the reliability of phenotypic information along the selection process. Large experimental errors, genotype by environment interaction, the influence of quality of planting material (and other epigenetic effects) and the changing nature of the association between key traits, such as FRY and DMC are some of the problems faced by cassava breeders, particularly when selecting in the large SRT. The use of breeding value of progenitors based on average SIN (proposed by (Ceballos et al., 2004)), seems to offer limited benefits (Ceballos et al., 2016). Results presented here provide feasible explanations for the conclusions reached in that “*sister*” article.

The use of HIN as indirect approach for improving FRY was disappointing. Based on this finding and the large expenses related to weighing aboveground biomass, the cassava breeding program at CIAT decided to no longer measure and use HIN in the selection process and rather rely on the easier to take PTS. However, a new term will be incorporated in the SIN equation. This new term will assess uniformity and vigor of sprouting stems 3–4 weeks after planting. This is a response to the challenge of climate change that require enhanced capacity of planting material to sprout, even after a longer-than-expected storage period (Ceballos et al., 2011).

Data presented in this study provides indirect evidence of the influence of the nutritional, physiological and health status of the planting material on the performance of the populations. It is this factor, along other potential epigenetic ones, that may explain the weak correlations between data in early vs. late stages of selection. Perhaps the magnitude of this problem may be reduced if seedling plants are grown in the target environment.

Selection based on SIN is proving effective improving simultaneously key traits, such as FRY, DMC, and PTS. The relationship among these variables, however, changes drastically through the selection process precisely because of selection. Phenotypic association between these variables, therefore, cannot be stated without defining first at what stage of the selection process their relationship is considered.

Unreliable phenotypic data in SRT is a major problem that may need to be addressed. Perhaps splitting the one-row plot (with 6–8 plants) into two reps (with 3–4 plants) may be an option. However, this would create considerable logistic problems and duplicate the volume of data generated. The omission of HIN in future harvests will simplify operations considerably, so the alternative of planting SRT in two replications may merit some consideration (if this is done, the name of this type of trial will have to be obviously changed). Improving the reliability of information in early stages of selection would be particularly relevant for genomic selection. The main advantage of this promising technology is to accelerate genetic gains by shortening each cycle of selection (2–3 years per cycle rather than 6–8 years in standard phenotypic recurrent selection). Higher quality and reliability of data from SRT would be highly beneficial for conventional and genomic selection in cassava.

## Author contributions

OJB: curated the data, consolidated it into a large file, and conducted most of the statistical analyses; JP: was an associate to the breeding program and curated and stored data year after year; JL: conducted the trials at the sub-humid environment; FC: is a senior associate of the program that helped in the overall activities of the program; NM: coordinated the production of segregating progenies and the seedling nurseries from which the planting material for the SRT was generated; LP: is an assistant in charge of data uploading and curation; CH is the coordinator of the program and also a senior cassava breeding. He reviewed and improved earlier versions of the manuscript. HC is the senior cassava breeder who implemented the changes in the breeding scheme and initiated the process to publish this article by asking the key questions it addresses. He wrote and corrected the different versions of the manuscript.

### Conflict of interest statement

The authors declare that the research was conducted in the absence of any commercial or financial relationships that could be construed as a potential conflict of interest.

## Footnotes

^1^Microsoft Corporation (2015). http://www.microsoft.com

## References

[B1] Benesi I. R. M. Labuschagne M. T. Herselman L. Mahungu N. (2010). Ethnobotany, morphology and genotyping of cassava germplasm from Malawi. J. Biol. Sci. 10, 616–623. 10.3923/jbs.2010.616.623

[B2] Bräutigam K. Vining K. J. Lafon-Placette C. Fossdal C. G. Mirouze M. Gutiérrez Marcos J.. (2013). Epigenetic regulation of adaptive responses of forest tree species to the environment. Ecol. Evol. 3, 399–415. 10.1002/ece3.461 23467802 PMC3586649

[B3] Burton G. W. (1992). Recurrent restricted phenotypic selection. Plant Breed. Rev. 9, 101–113. 10.1002/9780470650363.ch5

[B4] Byrne D. (1984). Breeding cassava. Plant Breed. Rev. 2, 73–134. 10.1002/9781118060995.ch3

[B5] Ceballos H. Fregene M. Pérez J. C. Morante N. Calle F. (2007). Cassava Genetic Improvement, in Breeding Major Food Staples, eds Kang M. S. Priyadarshan P. M. (Ames, IO: Blackwell Publishing), 365–391.

[B6] Ceballos H. Hershey C. Becerra-López-Lavalle L. A. (2012). New approaches to cassava breeding. Plant Breed. Rev. 36, 427–504. 10.1002/9781118358566.ch6

[B7] Ceballos H. Iglesias C. A. Pérez J. C. Dixon A. G. O. (2004). Cassava breeding: opportunities and challenges. Plant Mol. Biol. 56, 503–515. 10.1007/s11103-004-5010-5 15630615

[B8] Ceballos H. Okogbenin E. Pérez J. C. Becerra L. A. Debouck D. (2010). Cassava, in Root and Tuber Crops, ed Bradshaw J. (New York, NY: Springer Publishers), 53–96.

[B9] Ceballos H. Pérez J. C. Joaqui-B. O. Lenis J. I. Morante N. Hershey C.. (2016). Cassava breeding I: the value of breeding value. Front. Plant Sci. 7:1227. 10.3389/fpls.2016.01227 27621734 PMC5003041

[B10] Ceballos H. Ramirez J. Bellotti A. C. Jarvis A. Alvarez E. (2011). Adaptation of cassava to Changing Climates, in Crop Adaptation to Climate Change, eds Yadav S. Redden B. Hatfield J. L. Lotze-Campen H. (Hoboken, NJ: Wiley-Blackwell Publishers), 411–425.

[B11] CIAT (2001). Centro Internacional de Agricultura Tropical–CIAT, in Project IP3: Improved Cassava for the Developing World–Annual Report (Cali).

[B12] Gardner C. O. (1961). An evaluation of effects of mass selection and seed irradiation with thermal neutrons on yields of corn. Crop Sci. 1, 241–245. 10.2135/cropsci1961.0011183X000100040004x

[B13] Gonçalves Fukuda W. M. de Oliveira Silva S. Iglesias C. (2002). Cassava breeding. Crop Breed. Appl. Biotech. 2, 617–638. 10.12702/1984-7033.v02n04a18

[B14] Gonçalvez Fukuda W. M. Fukuda C. Leite Cardoso C. E. Lima Vanconcelos O. Nunes L. C. (2000). Implantação e evolução dos trabalhos de pesquisa participativa em melhoramento de mandioca no Nordeste Brasileiro. Documento CNPMF No. 92. Cruz das Almas: EMBRAPA.

[B15] Gonçalvez Fukuda W. M. Saad N. (2001). Participatory Research in Cassava Breeding with farmers in Northeastern Brazil. Document CNPMF 99. Cruz das Almas: EMBRAPA.

[B16] Grüneberg W. Mwanga R. Andrade M. Espinoza J. (2009). Breeding clonally propagated crops, in Plant Breeding and Farmer Participation, eds Ceccarelli S. Guimarães E. P. Weltzien E. (Rome, Italy: Food and Agriculture Organization of the United Nations), 175–322.

[B17] Hahn S. K. Terry E. R. Leuschner K. Akobundu I. O. Okali C. Lal R. (1979). Cassava improvement in Africa. Field Crops Res. 2, 193–226. 10.1016/0378-4290(79)90024-8

[B18] Hale I. L. Broders K. Iriarte G. (2014). AVavilovian approach to discovering crop-associated microbes with potential to enhance plant immunity. Front. Plant Sci. 5:492. 10.3389/fpls.2014.00492 25278956 PMC4167000

[B19] Hershey C. Henry G. Best R. Kawano K. Howeler R. Iglesias C. (2001). Cassava in Asia–Expanding the competitive edge in diversified markets, in A Review of Cassava in Asia with Country Case Studies on Thailand and Vietnam, (Rome, Italy: Food and Agricultural Organization of the United Nations – International Fund for Agricultural Development), 1–62.

[B20] Hershey C. Neate P. (2013). Eco-Efficiency: From Vision to Reality. Centro International de Agricultura Tropical (CIAT). CIAT Publication Number 381. Cali.

[B21] Howeler R. H. (2012). Recent trends in production and utilization of cassava in Asia, in The Cassava Handbook. A Reference Manual Based on the Asian Regional Cassava Training Course Held in Thailand, ed Howeler R. H. (Cali: CIAT Publication), 1–22.

[B22] Jennings D. L. Hershey C. (1985). Cassava breeding: a decade of progress from international programmes, in Progress in Plant Breeding, ed Russel G. E. (London: Butterworths Press), 89–116.

[B23] Jennings D. L. Iglesias C. A. (2002). Breeding for crop improvement, in Cassava: Biology, Production and Utilization, eds Hillocks R. J. Thresh J. M. Bellotti A. C. (Wallingford: CABI Publishing), 149–166.

[B24] Kawano K. (2003). Thirty years of cassava breeding for productivity–biological and social factors for success. Crop Sci. 43, 1325–1335. 10.2135/cropsci2003.1325

[B25] Kawano K. Cock J. H. (2005). Breeding cassava for underprivileged: institutional, socio-economic and biological factors for success. J. Crop Improv. 14, 197–219. 10.1300/j411v14n01_09

[B26] Kawano K. Goncalves Fukuda W. M. Cenpukdee U. (1987). Genetic and environmental effects on dry matter content of cassava root. Crop Sci. 27, 69–74. 10.2135/cropsci1987.0011183X002700010018x

[B27] Kawano K. Narintaraporn K. Narintaraporn P. Sarakarn S. Limsila A. Limsila J. (1998). Yield improvement in a multistage breeding program for cassava. Crop Sci. 38, 325–332. 10.2135/cropsci1998.0011183X003800020007x

[B28] Lebot V. (2010). Sweet potato, in Root and Tuber Crops, ed Bradshaw J. (New York, NY: Springer Publishing), 97–125.

[B29] Lenis J. I. Calle F. Jaramillo G. Pérez J. C. Ceballos H. Cock J. (2006). Leaf retention and cassava productivity. Field Crops Res. 95, 126–134. 10.1016/j.fcr.2005.02.007

[B30] Manu-Aduening J. A. Lamboll R. I. Ampong Mensah G. Lamptey J. N. Moses E. Dankyi A. A. (2006). Development of superior cassava cultivars in Ghana by farmers and scientists: the process adopted, outcomes and contributions and changed roles of different stakeholders. Euphytica 150, 47–61. 10.1007/s10681-006-9091-x

[B31] Morante N. Moreno X. Pérez J. C. Calle F. Lenis J. I. Ortega E. (2005). Precision of selection in early stages of cassava genetic improvement. J. Root Crops 31, 81–92.

[B32] Pérez J. C. Ceballos H. Ramirez I. C. Lenis J. I. Calle F. Morante N. (2010). Adjustment for missing plants in cassava evaluation trials. Euphytica 172, 59–65. 10.1007/s10681-009-0039-9

[B33] Quero-García J. Ivancic A. Lebot V. (2010). Taro and Cocoyam, in Root and Tuber Crops, ed Bradshaw J. (New York, NY: Springer Publishing), 149–172.

[B34] SAS (2008). SAS/STAT 9.1 User's Guide. Cary, NC: SAS Institute Inc.

[B35] Snedecor G. W. Cochram W. G. (1980). Statistical Methods 2nd Edn., Ames, IO: The Iowa State University Press.

[B36] Westwood N. N. (1990). Maintenance and storage: clonal germplasm. Plant Breed. Rev. 7, 111–128.

